# Beyond Mycorrhizae: A Multi‐Kingdom Community in Epiphytic Orchid Roots Reveals Cyanobacteria Intracellular and a Velamen Microbial Refuge

**DOI:** 10.1111/1758-2229.70434

**Published:** 2026-10-29

**Authors:** Eliana de Medeiros Oliveira, Edenilson Meyer, Paulo Emilio Lovato, Miguel Pedro Guerra, Tainara Gris, Anna Flávia Neri de Almeida, Juliana Lischka Sampaio Mayer

**Affiliations:** ^1^ LAMUME, Institute of Physics Federal University of Bahia (UFBA) Salvador Bahia Brazil; ^2^ LCME Federal University of Santa Catarina (UFSC) Florianópolis Santa Catarina Brazil; ^3^ Graduate Program in Plant Biology Universidade de Campinas (UNICAMP) Campinas São Paulo Brazil; ^4^ Graduate Program in Plant Genetic Resources Federal University of Santa Catarina (UFSC) Florianópolis Santa Catarina Brazil

**Keywords:** *Cattleya purpurata*, intracellular cyanobacteria, non‐mycorrhizal symbionts, orchid microbiome, symbiosis, velamen

## Abstract

Orchid biology has long been dominated by the paradigm of mycorrhizal dependency; however, the nutrient acquisition strategies of epiphytic orchids in canopy niches remain poorly understood. Using an integrated approach of multilocus metabarcoding (16S, ITS, 18S) and advanced microscopy (SEM, TEM, CLSM), we characterised the root microbial community of the threatened epiphytic orchid *Cattleya purpurata*. We report the discovery of intracellular *Nostoc‐*like cyanobacteria within cortical cells, coexisting with the mycorrhizal fungus *Tulasnella* at a root colonisation rate of 18%. Furthermore, we identified: (1) a diverse velamen‐associated microbiome hosting plant growth‐promoting bacteria (e.g., the *Burkholderia‐Caballeronia‐Paraburkholderia* complex), nematodes and microalgae; and (2) a vast unknown fungal diversity, with over 39 genera and numerous ASVs unmatched in databases. We demonstrate that *C. purpurata* roots sustain a complex multi‐kingdom community. We propose that the root velamen acts as a multi‐kingdom microbial refuge, expanding the concept of symbiosis in orchids beyond fungi. Based on the intracellular presence of *Nostoc‐*like cyanobacteria and their known diazotrophic capacity in other plant symbioses, we hypothesise a previously little‐known nitrogen pathway mediated by cyanobacteria in a canopy environment, to be functionally tested in future studies.

## Graphical abstract

This study redefines orchid ecology by revealing *Cattleya purpurata* roots host a multi‐kingdom microbiota. We report the first intracellular cyanobacteria in orchids, coexisting with mycorrhizal fungi in the cortex. The velamen functions as a structured refuge for specific, highly diverse microbial communities.

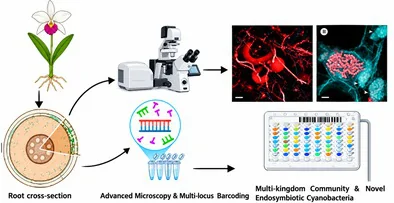

ChatGPT (OpenAI) was used only for visual enhancement of the graphical abstract, including resolution, brightness and contrast adjustments. The scientific content was created by the authors and remained unchanged.

## 1 Introduction

Plant–microbe symbioses have shaped the biosphere for hundreds of millions of years (Selosse et al. 2015), yet their roles in epiphytic plants, particularly those involving non‐fungal partners, remain underexplored. While mycorrhizal fungi are well‐characterised in orchids for their critical roles in seed germination and nutrient acquisition (Arditti and Pridgeon 2013; Dearnaley 2016), the ecological significance of bacterial and microeukaryotic symbionts is still poorly understood.

Recent evidence suggests that the velamen's unique architecture, with its thickened walls, pore networks and water‐retention capacity (Benzing 2008; Zotz and Winkler 2013), may harbour diverse microbial communities (Deepthi and Ray 2020). However, the taxonomic composition, spatial organisation and functional contributions of these communities remain unresolved.

Here, we investigate *Cattleya purpurata* (Lindl. & Paxton) Van den Berg, an epiphytic orchid endemic to the Atlantic Forest and classified as vulnerable (CNCFlora 2013). This orchid species is among the most affected by illegal collection in native ecosystems. It faces natural challenges related to pollination and the establishment of new generations (Caballero‐Villalobos et al. 2017), in addition to habitat loss caused by the expansion of urban and agricultural frontiers (Nascimento 2021). *Cattleya purpurata* has a distribution restricted to the Atlantic Forest of southern and southeastern Brazil, and its natural populations have undergone considerable decline in recent decades (CNCFlora 2013; Nascimento 2021).

Integrating advanced microscopy (SEM, TEM and Confocal Laser Scanning Microscopy—CLSM) with multilocus metabarcoding (16S rRNA, 18S, ITS), we map microbial distribution across root tissues and identify symbiotic interactions. We use the term ‘symbiosis’ in its broad classical sense, following de Bary, to refer to the intimate association between different species, regardless of whether the interaction is mutualistic, commensal or parasitic (de Bary 1879; Chapman and Margulis 1998, Paracer and Ahmadjian 2000).

We hypothesise that *C. purpurata* roots function as aerial microecosystems in which fungi, bacteria, photosynthetic microorganisms and microscopic animals form complex communities that may contribute to nutrient dynamics and ecological functioning in canopy habitats.

## 2 Experimental Procedures

### 2.1 Plant Material

Roots of *C. purpurata* were collected from natural populations in the Atlantic Forest in southern Santa Catarina Island, Brazil. The exact location of the populations is not provided because of the vulnerability of the species, but all collections were authorised under SISBIO licence no. 60177.

For morphological and metabarcoding analyses, roots were washed in running water and the velamen was gently scraped to reduce the presence of loosely attached epiphytes.

### 2.2 Micromorphological Analyses

#### 2.2.1 Confocal Laser Scanning Microscopy (CLSM)

Roots were fixed in 4% paraformaldehyde (SigmaAldrich) in 0.1 M sodium phosphate buffer (pH 7.2), prepared from sodium phosphate salts (SigmaAldrich). Fixed samples were frozen, and sections approximately 60 μm thick were obtained using a Leica CM1850 cryostat at LAMEB, Universidade Federal de Santa Catarina. Sections were mounted on slides. Part of the sections was mounted in water under a coverslip and examined for chlorophyll autofluorescence in order to detect photosynthetic microorganisms. The remaining sections were stained with acid fuchsin (Sigma‐Aldrich) for 1 min for general visualisation of microorganisms (Oliveira et al. 2026).

For CLSM imaging, orchid cell wall autofluorescence was recorded with excitation at 405 nm and emission at 420–480 nm (blue); chlorophyll autofluorescence was recorded with excitation at 488 nm and emission at 590–640 nm (orange–red); and acid‐fuchsin‐stained microorganisms were recorded with excitation at 514 nm and emission at 600–650 nm (red).

Images were acquired using a Leica SP5 confocal laser scanning microscope (Leica Microsystems, Wetzlar, Germany) at the Laboratório Central de Microscopia Eletrônica (LCME), Universidade Federal de Santa Catarina (UFSC). Image stacks were captured along the *z*‐axis (z‐step: 3 μm; z‐range: 40 μm) and used to reconstruct three‐dimensional projections of microbial distribution in the velamen and cortex. Image processing was performed with Leica LAS AF Lite software version 4.0 at the Laboratório Multiusuário de Estudos Biológicos (LAMEB), UFSC.

#### 2.2.2 Scanning Electron Microscopy (SEM) and Transmission Electron Microscopy (TEM)

Root samples were fixed in 3% glutaraldehyde (Electron Microscopy Sciences [EMS], Hatfield, Pennsylvania, USA) in 0.1 M sodium cacodylate buffer (pH 7.2) for 24 h. Samples were then post‐fixed in 1% osmium tetroxide (EMS) for 2 h and dehydrated through an ascending acetone series (30%–100%) (Sigma‐Aldrich, St. Louis, Missouri, USA).

For SEM, samples were dried in a critical point dryer (EM CPD030, Leica Microsystems, Vienna, Austria), coated with a 25 nm gold layer using an EM SCD500 high‐vacuum sputter coater (Leica Microsystems, Vienna, Austria) and examined with a JEOL JSM‐6390LV scanning electron microscope (Jeol Ltd., Tokyo, Japan) at LCME, UFSC.

For TEM, a separate set of root samples was infiltrated with SPURR resin (EMS) and polymerised at 70°C. Ultrathin sections were cut, mounted on copper grids, and contrasted with uranyl acetate (EMS) and lead citrate (EMS). Images were obtained using a JEOL JEM‐1011 transmission electron microscope (Jeol Ltd., Tokyo, Japan) at LCME, UFSC (Oliveira et al. 2026).

### 2.3 Mycorrhizal Colonisation

Cross‐sections of roots (*n* = 10, obtained from five individuals) were stained with acid fuchsin and evaluated for the proportion of cortical area occupied by pelotons, the coiled hyphal structures of orchid mycorrhizal fungi found inside parenchyma cells. Both intact and degraded pelotons were considered, and the percentage of mycorrhizal colonisation was estimated following González‐Chávez et al. (2018).

### 2.4 Metabarcoding and Microbial Community Identification

Six orchid individuals were sampled. Roots from two individuals were combined to form one biological replicate, resulting in three pooled samples for DNA extraction and sequencing.

Fresh roots were lyophilized prior to DNA extraction. Total DNA was extracted using the DNeasy PowerSoil Pro Kit (QIAGEN), following the manufacturer's instructions. Amplicon‐based metabarcoding was performed for the 16S rRNA, 18S rRNA and ITS regions to characterise bacteria, microeukaryotes and fungi, respectively. For 16S rRNA, the primers 515F (5′‐GTGCCAGCMGCCGCGGTAA‐3′) and 806R (5′‐GGACTACHVGGGTWTCTAAT‐3′) were used; this primer pair targets the V4 region, rather than the V3–V4 region. For ITS, the primers ITS1 (5′‐TCCGTAGGTGAACCTGCGG‐3′) and ITS2 (5′‐GCTGCGTTCTTCATCGATGC‐3′) were used. For 18S rRNA, the primer pair Euk1391F/EukBr was used; the corresponding sequences were 5′‐GTACACACCGCCCGTC‐3′ and 5′‐TGATCCTTCTGCAGGTTCACCTAC‐3′, respectively. This primer pair is commonly employed for the V9 region of 18S rRNA. During PCR amplification, peptide nucleic acid (PNA) clamps targeting host chloroplast and mitochondrial sequences were added to reduce non‐target amplification. Amplicons were visualised on 1.5% agarose gels, purified with AMPure XP Beads (Beckman Coulter, Life Sciences) and sequenced on an Illumina MiSeq platform using 2 × 250 bp paired‐end chemistry, with an average depth of 50,000 paired‐end reads per sample. All steps from DNA extraction to sequencing were performed by NGS Soluções Genômicas between September and October 2023.

Bioinformatic analyses were conducted in QIIME 2 version 2025.4, using the DADA2 plugin for quality filtering, denoising, merging of paired‐end reads and chimaera removal. Primer sequences were first removed using the qiime cutadapt trim‐paired plugin, and the trimmed reads were then processed with qiime dada2 denoise‐paired to generate representative amplicon sequence variants (ASVs). Taxonomic assignment of ASVs was performed with the qiime feature‐classifier classify‐sklearn plugin. For 16S rRNA and 18S rRNA data, the SILVA 138 database was used, whereas ITS data were classified against UNITE (release 2025). ASV abundance tables and taxonomic annotations were exported for downstream analyses, including bar plots and alpha‐ and beta‐diversity analyses.

## 3 Results

### 3.1 Compartmentalization of the Root Microbiome

We identified two distinct microbial niches in the roots of *C. purpurata* , the root cortex and the velamen.

The first niche is the root cortex, where pelotons, both intact and in various stages of digestion, were observed (Figure 1a). The ultrastructure of the hyphae of the pelotons, especially the presence of the dolipore, a morphologically specific septal structure of several Basidiomycota taxa, allowed us to determine the genus of the mycorrhizal fungus as *Tulasnella*. Although we recorded numerous pellets through imaging, the observed mycorrhization rate was moderate, 18% of the total cortical area.

**FIGURE 1:**
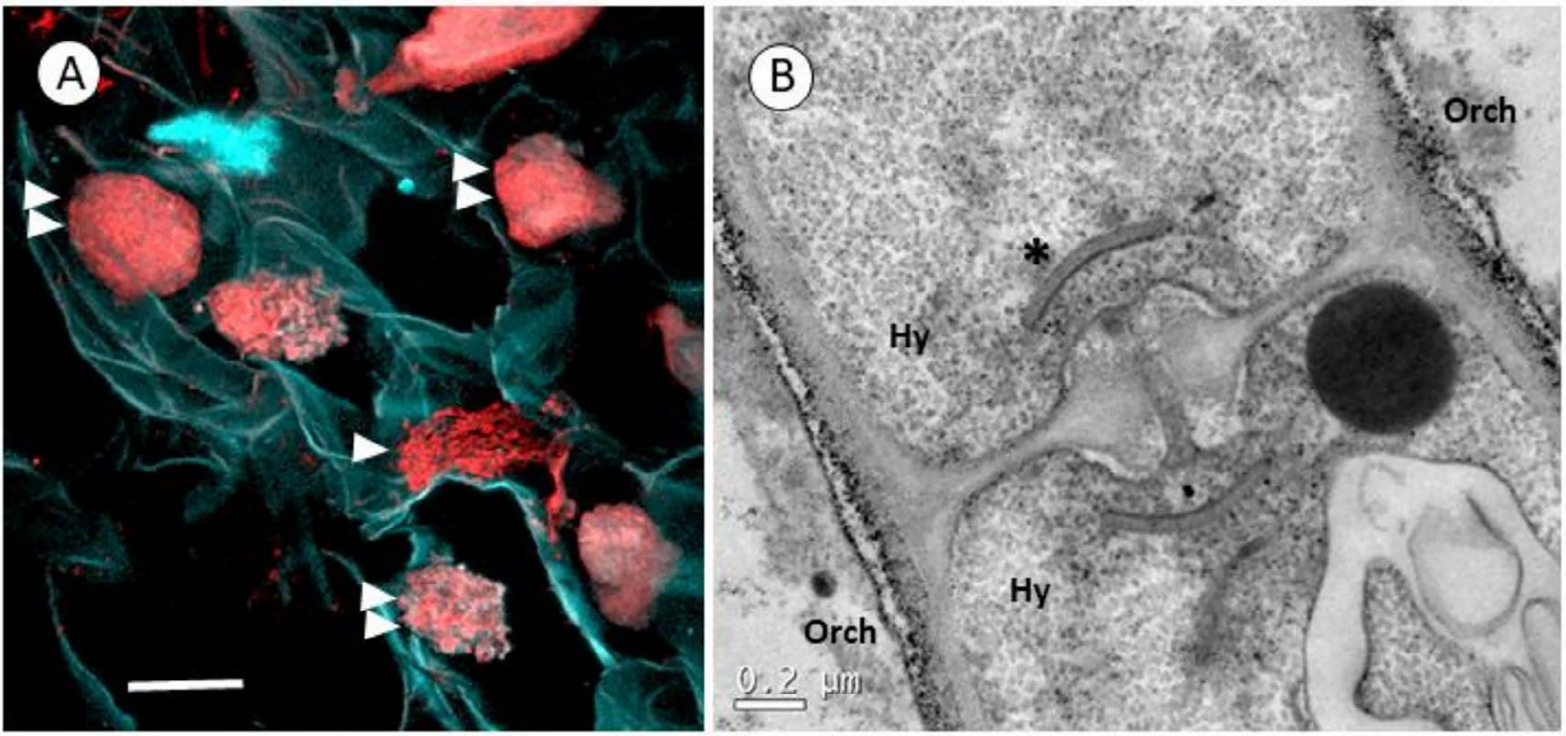
CSLM image (A) and TEM image (B) of pellets in *Cattleya purpurata* roots. (A) intact pellets (arrowhead) and degraded pellets (double arrowheads) in root cortex cells. In blue, the autofluorescence of the orchid cell wall; In red, hyphae stained with fuchsin. Scale bar: 50 μm. (B) Detail of the dolipore that allowed the identification of the mycorrhizal fungus genus. *Tulasnella*. Hy: hypha of the fungus *Tulasnella*; Orc: cortical cell of the orchid.

We recorded for the first time in orchids the presence of intracellular cyanobacteria in cortical tissue (Figure 2a). We observed cortical cells with intracellular cyanobacteria, similar in morphology and size to those of the genus *Nostoc*, alongside cells containing mycorrhizal pelotons (Figure 2b).

**FIGURE 2:**
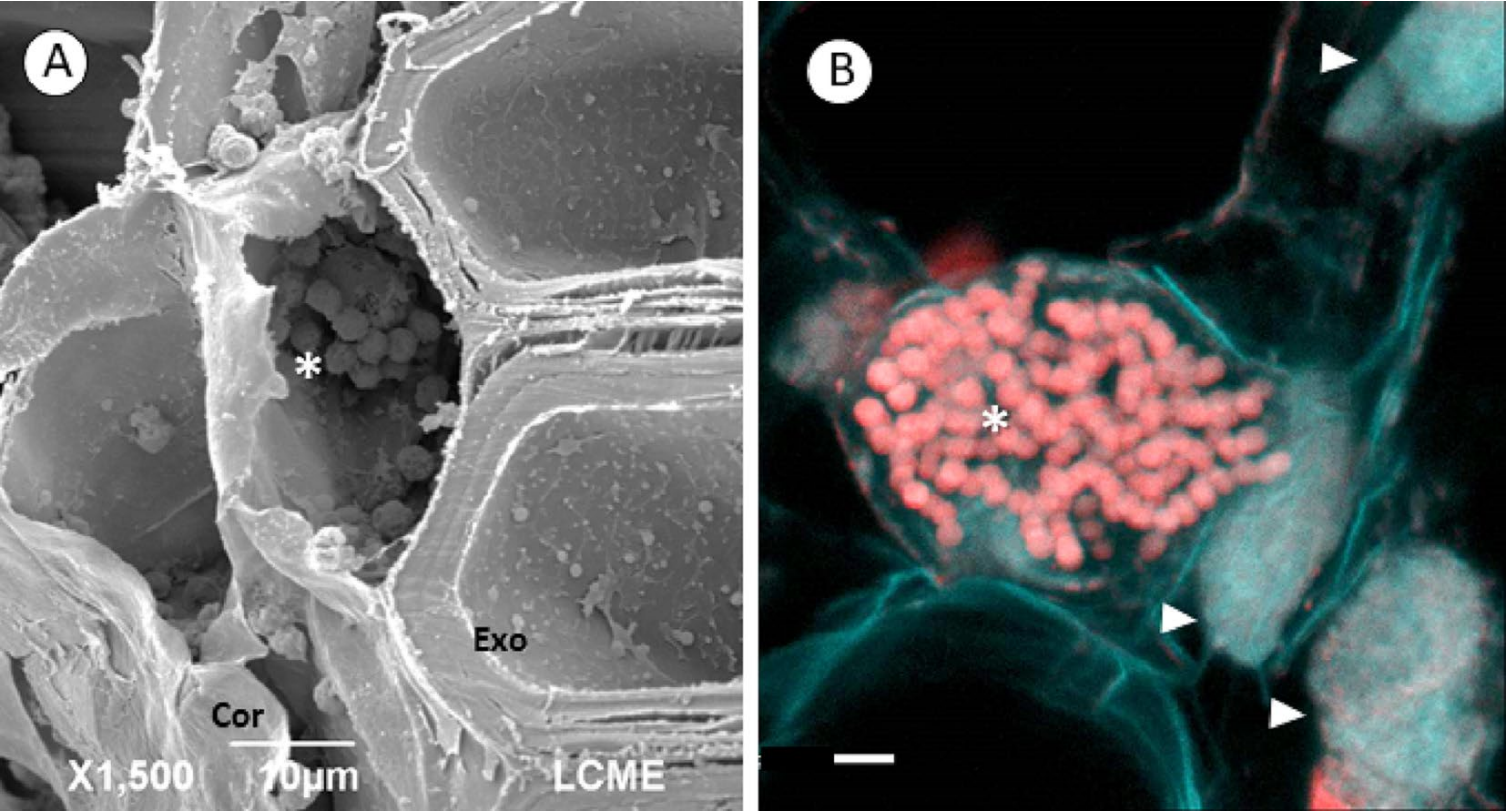
SEM image (A) and CLSM image (B) of intracellular cyanobacteria present in the cortical cells of *Cattleya purpurata*. In (A), image showing cyanobacteria (*) in cortical root cells. In (B), *Nostoc*‐like cyanobacteria (*), stained red with fuchsin, surrounded by the cortical cell wall (blue) and adjacent cells with degraded pelotons (arrowheads). Scale bar: 10 μm. Cor: Root cortex; Exo: Exodermis.

The second root niche is the velamen, which harbours not only clusters of cianobacteria (Figure 3a) but also nematode worms (Figure 3b) as well as green algae (Figure 3c).

**FIGURE 3:**
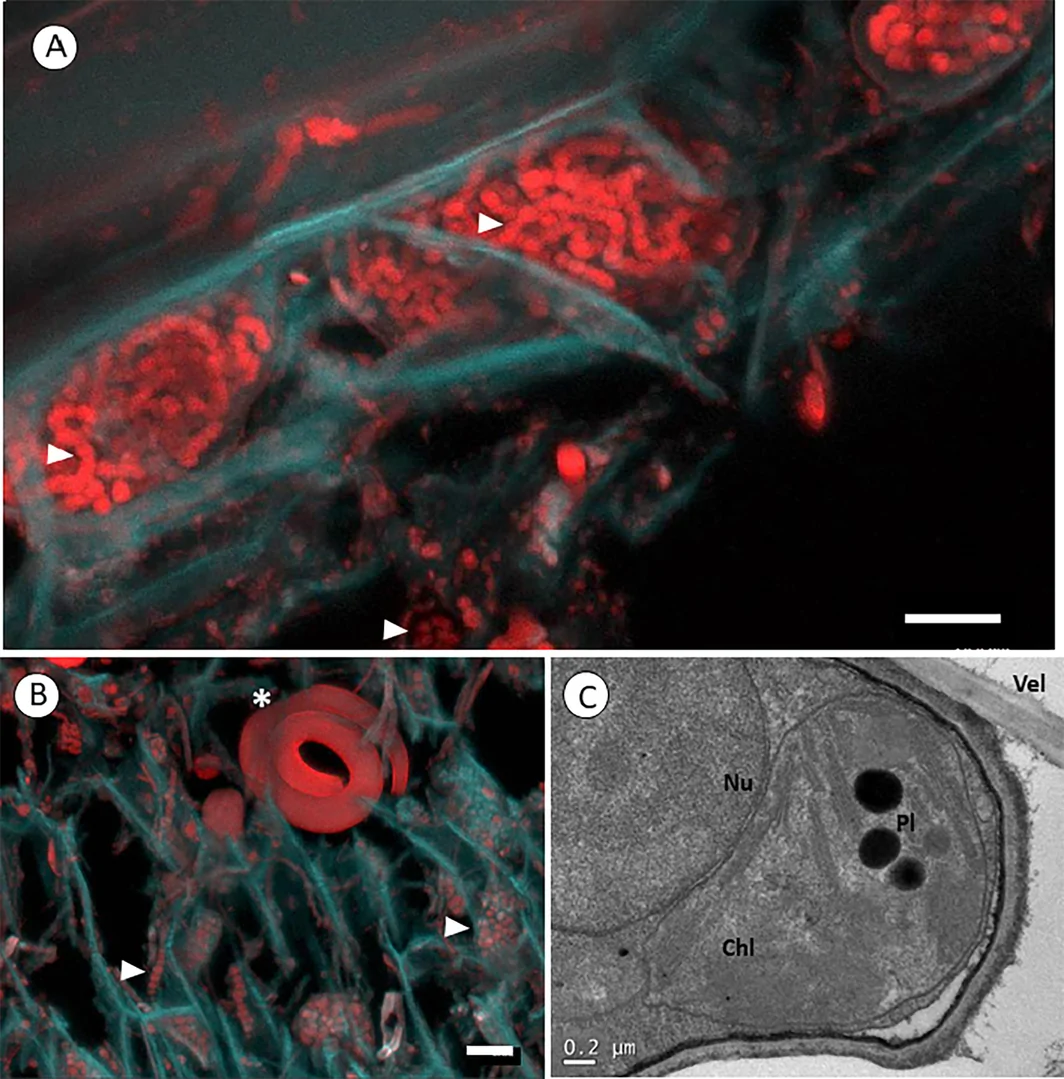
CLSM (A, B) and TEM (C) images showing the diversity of symbiotic microorganisms in the velamen of *Cattleya purpurata*. (A) Unstained section (mounted with water and coverslip only). Cluster of cyanobacteria (arrowhead) appear red due to chlorophyll autofluorescence. The velamen cell walls appear in blue. (B) Section stained with fuchsin. Velamen cell walls in blue; bacteria (arrowheads) and a nematode worm (*) in red. Scale bar: 20 μm. (C) Green algal cell, showing details of the chloroplast (Chl) with plastoglobules (Pl) and the cell nucleus (Nu). Vel: velamen cell wall.

### 3.2 Metabarcoding Reveals New Symbionts and High Species Richness

#### 3.2.1 Bacteria (16S V4)

The metabarcoding sequence of the 16S V4 region, aimed to identify bacteria, revealed 15 phyla, 163 genera and 513 ASVs (Amplicon Sequence Variants), demonstrating great bacterial richness in the roots of *C. purpurata* . Among the most abundant genera were bacteria not previously known as orchid endophytes, such as *Conexibacter* and *Acidothermus*, as well as taxa known as plant growth promoters, like Nocardioides, *Jatrophihabitans*, *Mucilaginibacter, Mycobacterium* and Eremiobacterota WPS‐2 (Figure 4).

**FIGURE 4:**
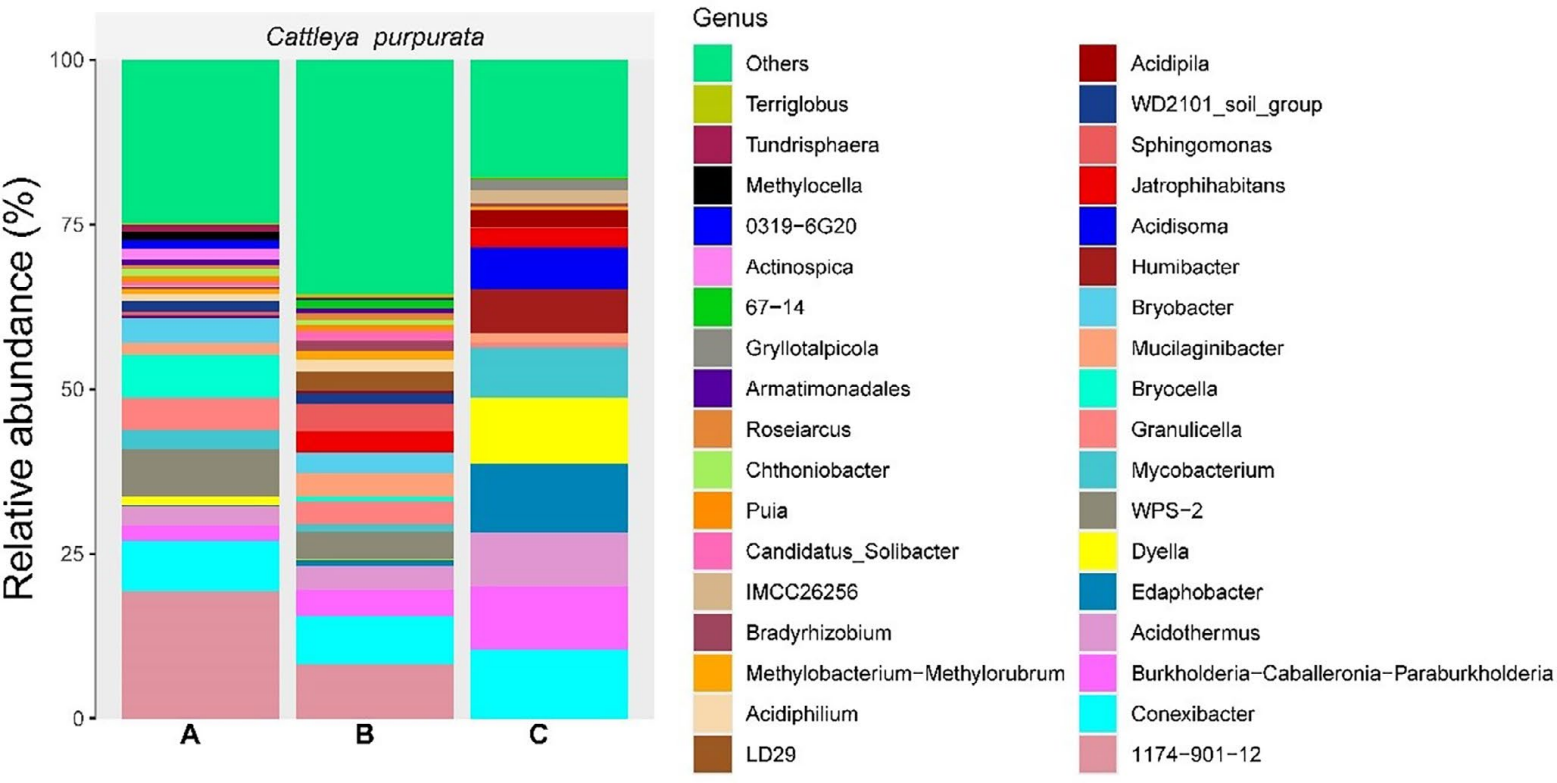
Relative abundance of bacterial microorganisms (16S rRNA gene sequencing) in roots of *Cattleya purpurata*. Each sample (A, B and C) represents a set of DNA extracted from roots of two distinct individuals, totaling six individuals analysed.

Proteobacteria constituted the most diverse and abundant bacterial phylum detected, comprising 193 ASVs and the highest cumulative count of reads across all three samples. Within this phylum, the genera standing out in terms of abundance are *Sphingomonas, Acidiphilium, Burkholderia‐Caballeronia‐Paraburkholderia* and *Methylobacterium‐Methylorubrum* (Figure 4).

#### 3.2.2 Microeukaryotes (18S Earth Project)

Our analyses revealed four distinct phyla of microeukaryotes associated with orchid roots. This study provides the first record of *Acanthamoeba* (Amoebozoa) as a possible symbiont in orchids and, to our knowledge, in any plant group (Figure 5).

**FIGURE 5:**
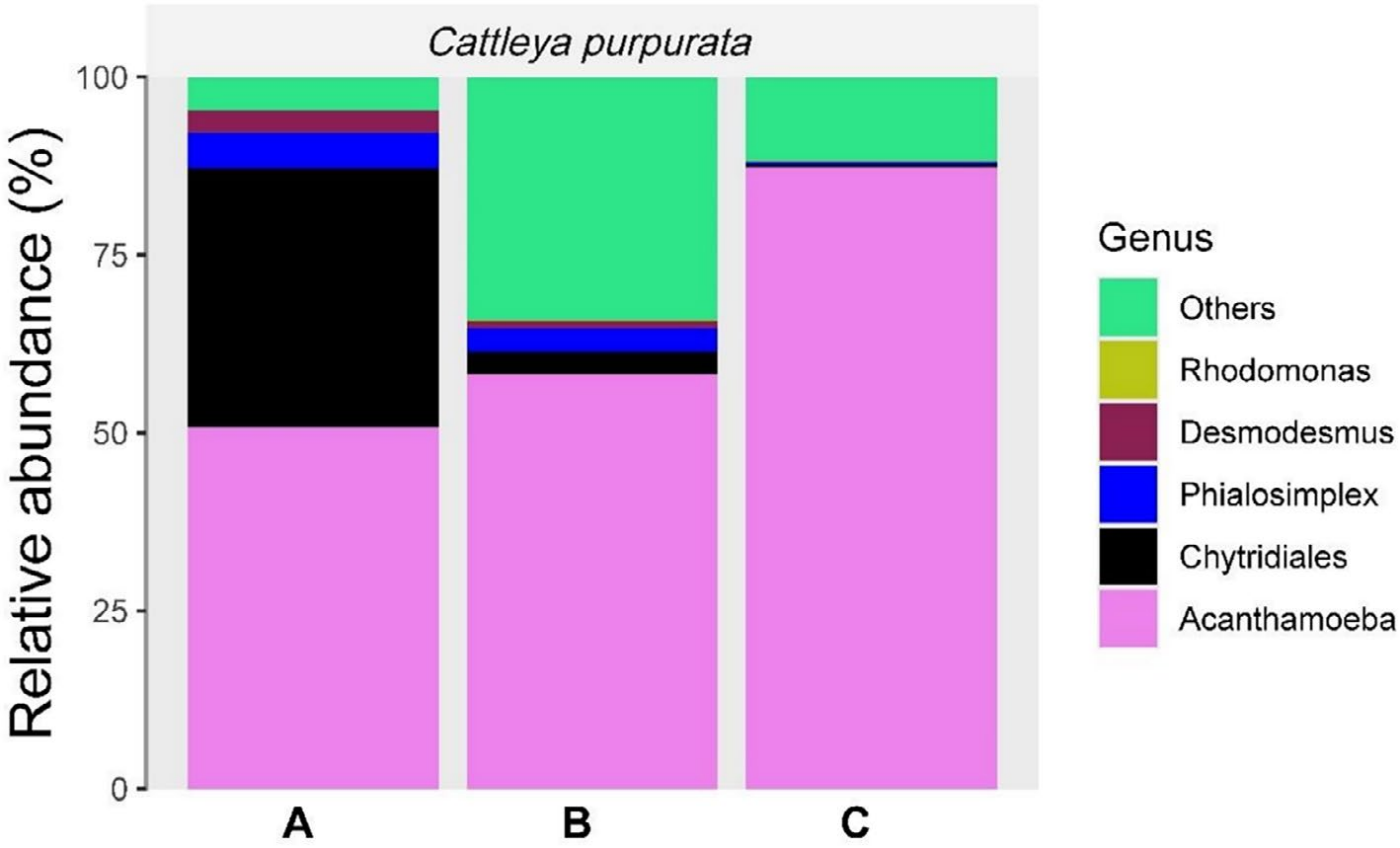
Relative abundance of eukaryotic microorganisms (18S rRNA gene sequencing) in roots of *Cattleya purpurata*. Each sample (A, B and C) represents a pool of DNA extracted from roots of two distinct individuals, totalling six individuals analysed.

A second fundamental finding was the detection of cryptophytes of the genus *Rhodomonas*, which also represent the first report of this group as orchid symbionts (Figure 5). These results broaden the known diversity of microbial eukaryotes in orchid root systems and suggest previously unrecognised ecological interactions.

#### 3.2.3 Fungi (ITS)

For the ITS region, most of the 248 ASVs generated could not be associated with sequences in gene databases (Figure 6). Ascomycete fungi were the most diverse and abundant fungi in roots, with *Hypomyces, Teratosphaeria* and *Paradevriesia* as the most frequent genera. Curiously, traditional orchid mycorrhizal fungi from the large paraphyletic *Rhizoctonia*‐like group were not sampled by ITS‐region sequencing (Figure 6).

**FIGURE 6:**
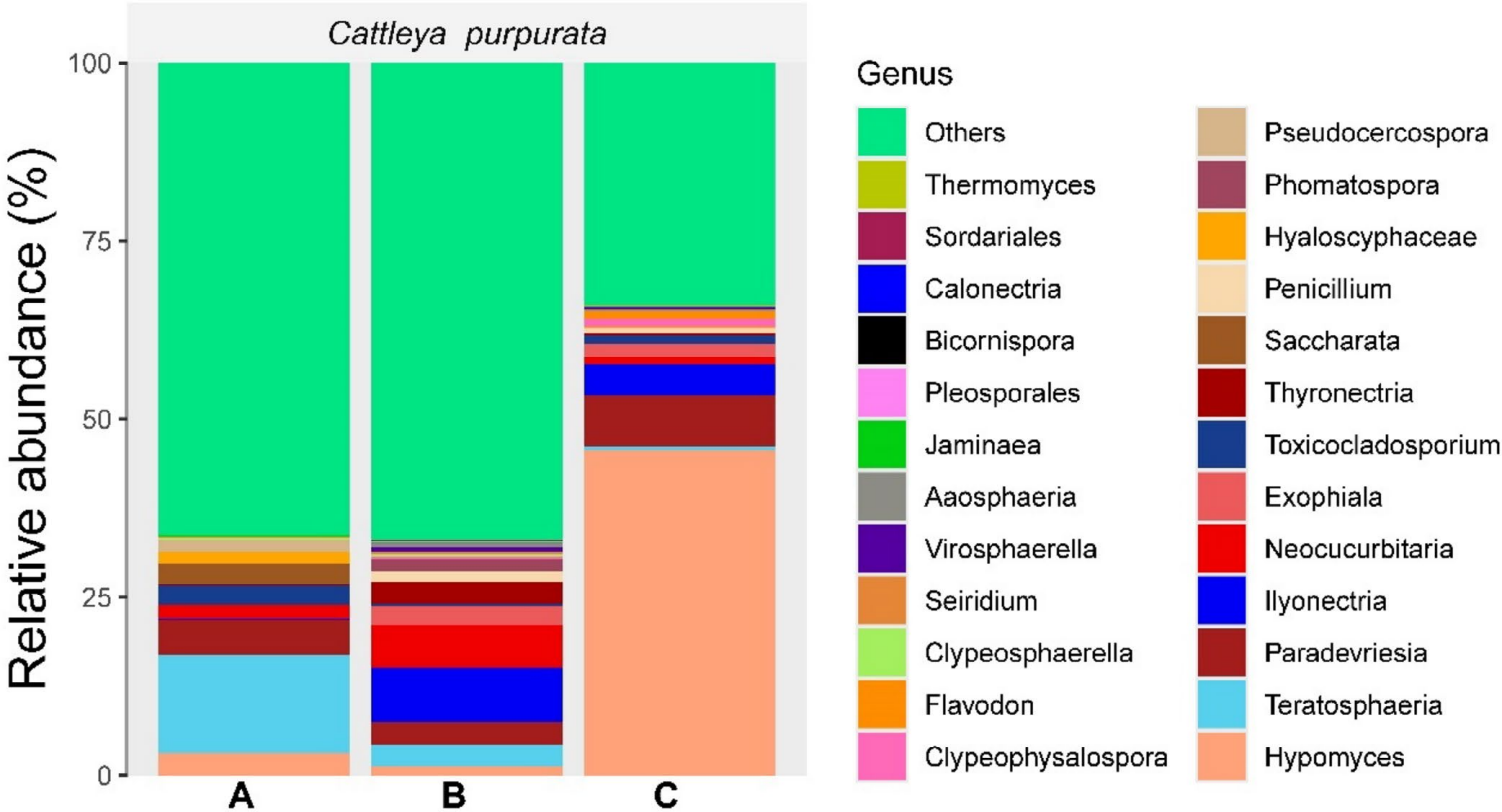
Relative abundance of fungal microorganisms (ITS gene sequencing) in roots of *Cattleya purpurata*. Each sample (A, B and C) represents a DNA pool extracted from roots of two distinct individuals, totaling six individuals analysed.

The supplementary data provide a comprehensive portrait of the microecosystem in the aerial roots of C*attleya purpurata*. Imaging of the orchid root sections, including 3D reconstructions using confocal laser scanning microscopy (CLSM) and scanning electron microscopy (SEM) analyses (Videos S1, S2 and S3; and Figures S1—S5), provided complementary visual evidence of the spatial organisation of the microbiota in the roots. Furthermore, imaging of unstained root sections allowed us to visualise photosynthetic microorganisms (algae and cyanobacteria) based on their chlorophyll autofluorescence (Figures S6–S8).

## 4 Discussion

### 4.1 Cyanobacteria in Cortical Cells: A New Nitrogen Pathway in Orchids?

The presence of intracellular cyanobacteria in cortical cells of *Cattleya purpurata* suggests that nutrient acquisition in this orchid may be more complex than a strictly fungus‐mediated strategy.

Orchid mycorrhizal fungi are recognised as key partners in germination, establishment and nutrient transfer, including nitrogen and phosphorus acquisition (Arditti 1992; Cameron et al. 2006, 2007; Dearnaley et al. 2012; Fochi et al. 2017). However, although our metabarcoding data did not detect *Nostoc* spp., the morphology of the observed cyanobionts is consistent with filamentous members of the Nostocales, an order that includes endophytic and often diazotrophic species that form intracellular associations with plants (Álvarez et al. 2020, 2023). These microorganisms could therefore represent an additional nitrogen source, should biological N2 fixation be confirmed. This possibility is particularly relevant given the relatively low rate of mycorrhizal colonisation observed in *C. purpurata* roots (18%), compared to rates reported for other orchids, which can exceed 80% (Sathiyadash et al. 2012). The evidence provided in the present study is structural rather than functional: the proximity between cyanobacteria and fungal pelotons supports the hypothesis of a possible tripartite association, but we did not seek to demonstrate nutrient exchange among the partners. Functional confirmation will require direct physiological approaches, notably ^15^N_2_ incorporation coupled with isotopic tracking in orchid tissues.

In angiosperms, records of symbiotic associations with cyanobacteria are uncommon (Rai et al. 2000; Deepthi and Ray 2018; Tsavkelova et al. 2022; de Vries and de Vries 2022; Álvarez et al. 2023). Even rarer are records of intracellular cyanobacteria, which have only been found in the genus *Gunnera* (Khamar et al. 2010) and in *Oryza* (Álvarez et al. 2023).

### 4.2 Velamen: A Microbial Biodiversity Hotspot

The architecture of the velamen, characterised by multiple cell layers, empty air‐filled spaces and thickened walls with many pores, appears to be a structured microhabitat retaining water, dissolved nutrients and microorganisms inside the aerial roots (Benzing et al. 1983; Zotz and Winkler 2013). In *C. purpurata* , microscopy revealed a diverse microbial assemblage spanning multiple trophic levels, from primary producers (cyanobacteria) to potentially bacterivorous and fungivorous nematodes. Rather than viewing the velamen as merely a passive absorptive tissue, these observations support its role as an ecologically dynamic interface between the orchid root and the surrounding environment. This interpretation aligns with previous studies showing that the velamen of epiphytic orchids can harbour diverse fungal communities, including ascomycetes that remain spatially segregated from intracellular orchid mycorrhizal fungi (Herrera et al. 2010; Pujasatria et al. 2022), as well as complex bacterial communities that vary across orchid organs, species and ecological contexts (Li et al. 2017; Zhu et al. 2022; Andrade et al. 2023; Mahadeo et al. 2024).

Among the bacterial taxa detected in the velamen, *Mycobacterium* and *Edaphobacter* were among the most abundant. Although their ecological roles in epiphytic orchids remain unknown, members of these genera have been documented as plant growth promoters, with functions including phosphate solubilisation and abiotic stress tolerance (Madhaiyan et al. 2010; Lee and Cho 2024).

The predominance of Proteobacteria across all three samples, the most diverse and abundant phylum recovered in the 16S dataset (193 ASVs), deserves attention in a functional context. Within this phylum, *Sphingomonas* stands out as a genus with documented direct plant growth‐promoting activity in orchids: *S. paucimobilis* ZJSH1, isolated from *Dendrobium officinale*, promotes orchid growth via phytohormone secretion (IAA, gibberellins, zeatin, salicylic acid) and nitrogen fixation confirmed by nifH gene sequencing (Yang et al. 2014). Similarly, members of the *Burkholderia‐Caballeronia‐Paraburkholderia* complex, also well represented in our dataset, have been isolated as nitrogen‐fixing endophytes from *Cymbidium* sp. orchid roots (Gontijo et al. 2018) and diazotrophic endophytes were reported across multiple genera from roots and leaves of the congener *Cattleya walkeriana* (Andrade et al. 2023). Taken together, these observations suggest that Proteobacteria may contribute meaningfully to the nitrogen balance of *C. purpurata* , though functional confirmation through nifH gene sequencing or ^15^N_2_ incorporation experiments is still required.

ITS‐region analyses showed a high diversity of ascomycete fungal genera, and among them, a wide variety of ecological roles, including the taxon *Hypomyces*, which parasitizes other fungi (Douhan and Rizzo 2003; Szumigaj‐Tarnowska and Szczechura 2024), *and Teratosphaeria* and *Saccharata*, which are opportunistic pathogens in other angiosperm species (Slippers et al. 2013; Silva et al. 2020; Havenga et al. 2021). In contrast, orchid mycorrhizal fungi of the *Rhizoctonia*‐like group were not sampled in the sequencing. It is worth noting that sequencing of the ITS region, the most widely used marker for fungal diversity (Grantham et al. 2015; Banerjee et al. 2024; Tedersoo et al. 2014), did not detect the genus *Tulasnella*, revealing methodological limitations.

The genus*Tulasnella* (anamorph *Epulorhiza*) was identified by its diagnostic dolipore ultrastructure, characterised by curved, imperforate parenthesomes and an electron‐dense region proximal to the pore (Currah and Sherburne 1992; Moore 1996).

Finally, a substantial proportion of fungal ASVs could not be confidently assigned to named taxa, underscoring the incompleteness of current reference databases for fungi associated with Neotropical epiphytic orchids. Other studies on epiphytic orchids from Ecuador and China similarly reported high fungal richness alongside a large number of unresolved taxa (Herrera et al. 2010; Cevallos et al. 2018, 2022; Wang et al. 2021). Faced with this gap, the Kew Gardens Museum has initiated an ambitious project to sequence its entire mycological collection, significantly expanding gene sequences in available fungal gene banks.

In a pioneering approach, this study successfully combined morphological and molecular tools to identify previously unrecorded microorganisms living in association with orchid roots, including nematode worms and the amoebozoan *Acanthamoeba*. Furthermore, we detected aquatic protists such as cryptophyte microalgae of the genera *Rhodomonas* and *Desmodesmus*, groups rarely recorded in plant‐associated systems (Arguelles 2025). These findings highlight the role of the velamen as a humid microhabitat that sustains microbial diversity, including aquatic taxa, even in aerial/canopy environments.

## 5 Conclusions

Our results suggest that interactions within orchid roots are far more complex and diverse than traditionally assumed, raising questions about the scale of the symbiotic networks involved; the dynamics of energy flow within these systems; and the dispersal mechanisms that allow aquatic microorganisms to colonise aerial roots.

This work opens new issues for research on the evolution of symbioses in epiphytic habitats, positioning the velamen as an unexplored hotspot of microbial biodiversity. Future studies should prioritise: (I) functional analyses of these interactions (e.g., metabolic exchanges, microfood web dynamics and host benefits); (II) confirmation of nitrogen fixation and its availability to orchids using direct physiological approaches, such as ^15^N_2_ incorporation coupled with isotopic tracking in orchid tissues; and (III) broader sampling across orchid taxa and ecosystems to assess microbial prevalence and environmental drivers.

## Author Contributions

**Eliana de Medeiros Oliveira:** conceptualization, methodology, data curation, investigation, validation, formal analysis, visualization, writing – original draft, writing – review and editing. **Edenilson Meyer:** conceptualization, methodology, software, data curation, investigation, formal analysis, visualization, writing – original draft, writing – review and editing. **Paulo Emilio Lovato:** conceptualization, methodology, data curation, investigation, formal analysis, supervision, writing – original draft, writing – review and editing. **Miguel Pedro Guerra:** conceptualization, methodology, data curation, investigation, supervision, formal analysis, writing – original draft, writing – review and editing. **Tainara Gris:** investigation, formal analysis, writing – original draft, writing – review and editing, conceptualization. **Anna Flávia Neri de Almeida:** methodology, investigation, formal analysis, writing – original draft, writing – review and editing. **Juliana Lischka Sampaio Mayer:** conceptualization, methodology, data curation, investigation, formal analysis, writing – original draft, writing – review and editing, supervision.

## Funding

The authors have nothing to report.

## Conflicts of Interest

The authors declare no conflicts of interest.

## Supporting information


**Video S1:** Detailed view of velamen spaces (cell walls fluorescing blue) with diverse cyanobacteria and fungal hyphae (fluorescing red).
**Video S2:** Velamen (cell walls fluorescing blue) with a nematode and fungal hyphae (both fluorescing red).
**Figure S2:** Unidentified microorganism (*) fluorescing bright red; velamen cell walls fluorescing bright blue. Scale bar: 30 μm.
**Figure S3:** Unidentified microorganisms (*), fungal hyphae (arrowheads) and bacteria (arrows) fluorescing bright red; velamen cell walls fluorescing bright blue. Scale bar: 30 μm.
**Video S3:** 3D‐rendered animation derived from Z‐stack imaging of the velamen, highlighting blue‐fluorescing cell walls and red‐fluorescing unidentified microorganisms (morphologically similar to *Caenorhabditis elegans* eggs).
**Figure S4:** SEM image showing a cross‐section of the orchid *Cattleya purpurata* root, displaying the velamen (Vel) and root cortex (exodermis: EXO, and cortical parenchyma: COR). Fungal hyphae (arrows) and degraded pelotons (*) are visible.
**Figure S5:** SEM image showing a cross‐section of *Cattleya purpurata* orchid root, displaying the velamen (Vel) and root cortex (exodermis: EXO, and cortical parenchyma: COR). The image reveals abundant velamen wall thickenings (arrows) and pores (*).
**Figure S6:** Confocal laser scanning microscopy (CLSM) image of unstained root sections. Algae and cyanobacteria appear in red (chlorophyll autofluorescence), and velamen cell walls appear in blue (intrinsic autofluorescence). Scale bar Escala: 30 μm.
**Figure S7:** Confocal laser scanning microscopy (CLSM) image of unstained root sections. Algae and cyanobacteria appear in red (chlorophyll autofluorescence), and velamen cell walls appear in blue (intrinsic autofluorescence). Scale bar Escala: 20 μm.
**Figure S8:** Confocal laser scanning microscopy (CLSM) image of unstained root sections. Algae and cyanobacteria appear in red (chlorophyll autofluorescence), and velamen cell walls appear in blue (intrinsic autofluorescence). Scale bar: 20 μm.

## Data Availability

The nucleotide sequences generated in this study have been deposited in the GenBank database under accession numbers: The GenBank NCBI accession numbers for the 16S rRNA are SUB15449814 (PV917733–PV918055). The GenBank NCBI accession numbers for the 18S Rrna is SUB15449861 (PX061018–PX061164). The GenBank NCBI accession number for the ITS rRNA is SUB15449881 (PV983001–PV983248). Complementary image data (additional micrographs and analyses) are available as Supplementary Files accompanying this publication.
