# Supplementary material for: Beyond Mycorrhizae: A Multi‐Kingdom Community in Epiphytic Orchid Roots Reveals Cyanobacteria Intracellular and a Velamen Microbial Refuge
Source: Environ Microbiol Rep. 2026 Oct 29;18(6):e70434. doi: 10.1111/1758-2229.70434 (PMC13720241; doi:10.1111/1758-2229.70434)
Supplement: Supplementary file 1 [file EMI4-18-e70434-s001.docx]

The nucleotide sequences generated in this study have been deposited in the GenBank database under accession numbers:

The GenBank NCBI accession numbers for the 16S rRNA is SUB15449814 (PV917733-PV918055).

The GenBank NCBI accession numbers for the 18S Rrna is SUB15449861 (PX061018 - PX061164).

The GenBank NCBI accession number for the ITS rRNA is SUB15449881 (PV983001 - PV983248).

The supplementary data provide a comprehensive portrait of the microecosystem in the aerial roots of *Cattleya purpurata*. Imaging of the orchid root sections, including 3D reconstructions using confocal laser scanning microscopy (CLSM) and scanning electron microscopy (SEM) analyses (Videos S1, S2 and S3; and Figs. S1 - S5), provided complementary visual evidence of the spatial organization of the microbiota in the roots. Furthermore, imaging of unstained root sections allowed us to visualize photosynthetic microorganisms (algae and cyanobacteria) based on their chlorophyll autofluorescence (Figures S6, S7 and S8).

**Supplementary Files**

Functional Imaging and 3D Reconstructions (from Confocal Laser Scanning Microscopy - CLSM):

- Video S1: Detailed view of velamen spaces (cell walls fluorescing blue) with diverse cyanobacteria and fungal hyphae (fluorescing red).

<https://drive.google.com/file/d/1Jv2mebv8kcZEBGwmDufjuRmukaH3AV9d/view?usp=sharing>

- Video S2: Velamen (cell walls fluorescing blue) with a nematode and fungal hyphae (both fluorescing red).

<https://drive.google.com/file/d/12BTkErig-c91JQ53WLfjpMniJESy-JEP/view?usp=sharing>

Morphologically unidentified microorganisms in the velamen of roots of the orchid *Cattleya purpurata* (Figs. S1, S2, and S3, and Video S3):

• Figure S1: Unidentified microorganism fluorescing bright red; velamen cell walls fluorescing bright blue. Scale bar: 15 µm.

- Figure S2: Unidentified microorganism (*) fluorescing bright red; velamen cell walls fluorescing bright blue. Scale bar: 30 µm.

- Figure S3: Unidentified microorganisms (*), fungal hyphae (arrowheads), and bacteria (arrows) fluorescing bright red; velamen cell walls fluorescing bright blue. Scale bar: 30 µm..

- Video S3: 3D-rendered animation derived from Z-stack imaging of the velamen, highlighting blue-fluorescing cell walls and red-fluorescing unidentified microorganisms (morphologically similar to *Caenorhabditis elegans* eggs):

https://drive.google.com/file/d/1QMA5HNJv-Tan4JaymCJMUVj32YH5BXNf/view?usp=sharing

SEM images showing velamen architecture, with pores and wall thickenings, creating a shelter environment for microorganisms (Figs. S4 and S5).

- Figure S4: SEM image showing a cross-section of the orchid *Cattleya purpurata* root, displaying the velamen (Vel) and root cortex (exodermis: EXO, and cortical parenchyma: COR). Fungal hyphae (arrows) and degraded pelotons (*) are visible.

- Figure S5: SEM image showing a cross-section of *Cattleya purpurata* orchid root, displaying the velamen (Vel) and root cortex (exodermis: EXO, and cortical parenchyma: COR). The image reveals abundant velamen wall thickenings (arrows) and pores (*).

Figure S6: Confocal laser scanning microscopy (CLSM) image of unstained root sections. Algae and cyanobacteria appear in red (chlorophyll autofluorescence), and velamen cell walls appear in blue (intrinsic autofluorescence). Scale bar Escala: 30 µm.

Figure S7: Confocal laser scanning microscopy (CLSM) image of unstained root sections. Algae and cyanobacteria appear in red (chlorophyll autofluorescence), and velamen cell walls appear in blue (intrinsic autofluorescence). Scale bar Escala: 20 µm.

Figure S8: Confocal laser scanning microscopy (CLSM) image of unstained root sections. Algae and cyanobacteria appear in red (chlorophyll autofluorescence), and velamen cell walls appear in blue (intrinsic autofluorescence). Scale bar: 20 µm.
